# The Chinese version of the cognitive, affective, and somatic empathy scale for children: Validation, gender invariance and associated factors

**DOI:** 10.1371/journal.pone.0195268

**Published:** 2018-05-07

**Authors:** Jianghong Liu, Xin Qiao, Fanghong Dong, Adrian Raine

**Affiliations:** 1 University of Pennsylvania School of Nursing, Philadelphia, PA, United States of America; 2 University of Maryland, College Park, College of Education, College Park, MD, United States of America; 3 Hebei University School of Nursing, Baoding, Hebei, P.R.China; 4 University of Pennsylvania Departments of Criminology, Psychiatry, and Psychology, Philadelphia, PA, United States of America; University of Florence, ITALY

## Abstract

**Objectives:**

Empathy is hypothesized to have several components, including affective, cognitive, and somatic contributors. The only validated, self-report measure to date that assesses all three forms of empathy is the Cognitive, Affective, and Somatic Empathy Scale (CASES), but no current study has reported the psychometric properties of this scale outside of the initial U.S. sample. This study reports the first psychometric analysis of a non-English translation of the CASES.

**Methods:**

Confirmatory factor analysis was used to assess the factor structure of CASES as well as its associations with callous-unemotional traits in 860 male and female children (mean age 11.54± .64 years) from the China Jintan Child Cohort Study.

**Results:**

Analyses supported a three-factor model of cognitive, affective, and somatic empathy, with satisfactory fit indices consistent with the psychometric properties of the English version of CASES. Construct validity was established by three findings. First, females scored significantly higher in empathy than males. Second, lower scores of empathy were associated with lower IQ. Third, children with lower empathy also showed more callous-unemotional attributes.

**Conclusions:**

We established for the first time cross-cultural validity for Cognitive, Affective, and Somatic Empathy Scale (CASES). Our Chinese data supports the use of this new instrument in non-Western samples, and affirms the utility of this instrument for a comprehensive assessment of empathy in children.

## Introduction

Empathy has become an increasingly popular target of study for understanding neurobiological mechanisms underlying child aggression, antisocial behavior, and future conduct problems [1,2]. There is conflicting evidence from systematic literature reviews and meta-analyses regarding the association between empathy and externalizing behaviors, with some studies reporting only small or moderate negative correlations and others finding large effects [3–5]. Some authors have suggested that part of the variability in these findings can be attributed to differences in the measurement of empathy, as many researchers continue to operationalize empathy as a single global construct [1]. However, since the 1980’s, several multidimensional models of empathy have been proposed, including ones positing that empathy should be divided into cognitive, motor and affective components [4,6–8].

Cognitive empathy (drawn from theory-of-mind perspectives) reflects mental awareness of another person’s internal state, while affective empathy reflects the ability to experience another person’s internal state vicariously [2]. Motor empathy encompasses the subconscious mirroring behaviors one engages in, such as mimicking facial expressions, gestures, or body posturing, which serve as a vehicle for emotional contagion [2]. Further differentiation of empathy into positive and negative forms considers the sharing and vicarious experience of others’ negative and positive emotions [9]. This distinction has been understudied but is important as each form of empathy selectively activates different areas of the brain associated with negative and positive emotion. Furthermore, negative and positive empathy may be correlated with different outcomes [10], such as positive empathy being associated with prosocial behaviors and well-being [9]. Assessment of different forms of empathy have typically either been unidimensional or largely concentrated on cognitive and affective components. Yet even with the growing acceptance of empathy as a multifactorial phenomenon, quantitative data from psychometric studies inadequately provide empirical support for the differentiation of empathy into these three components. Such evidence could be useful for validating the measurement of empathy and disentangling previous conflicting findings by allowing researchers to parse the differential contributions of various forms of empathy to internalizing and externalizing conditions.

In an attempt to address this problem, Raine and Chen [11] recently developed a novel self-report instrument for assessing cognitive, affective, and somatic (motor) forms of positive and negative affect empathy in children. The intention was to create an instrument (termed CASES—the Cognitive, Affective, and Somatic Empathy Scale) that could globally capture various facets of empathy in a single assessment tool in order to inform better understanding of how empathy is manifested differentially across the lifespan and in relation to child development and mental health outcome. The CASES model is one among several multidimensional models of empathy that have been proposed since the 1970’s [6,12,13]. Confirmatory factor analyses supported a two-factor (positive and negative affect empathy) and three-factor (cognitive, affective, and somatic empathy) structure. Second-order factor models indicated the cognitive, affective, and somatic model could be differentiated into positive and negative forms of empathy. Internal consistency for the total empathy scale score was high (.91), and internal reliability for the three domains of cognitive, affective, and somatic empathy, as well as positive and negative empathy domains also proved satisfactory (α = .78-.84) [11]. All empathy domains were negatively correlated with externalizing behaviors but not internalizing conditions [11]. Lower empathy scores at baseline were associated with the presence of child- and parent-reported callous-unemotional (CU) traits at a one year follow-up. The authors suggest further research on empathy and child behavior and mental health outcomes could benefit from the inclusion of the CASES questionnaire and its novel and thorough investigation of less-studied aspects of empathy (somatic empathy, positive-negative affect empathy) [11].

As such, the purpose of this study is to examine the validity and reliability of a Chinese translation of the CASES measure. Psychometric analyses of empathy assessments to date have largely focused on their validation in Western samples, but investigation into other populations is warranted given the universality of empathy as a common human experience. Expressions of emotion and interpersonal behaviors are culture-bound, underscoring the need for translations of these assessment tools to account for cross-cultural diversity in empathy as a construct and to accurately capture its presence and correlates in non-Western populations. Valid and reliable assessments for empathy in Chinese youth are lacking and would be valuable for research to better understand risk factors and potential interventions for child internalizing and externalizing behaviors. Furthermore we are aware of only two studies to date [11,14] that have investigated empathy using a single tool to capture cognitive, affective, and motor components collectively. Thus, the current study represents a novel contribution to the literature in two ways: by providing the first non-English version of a newly validated empathy measure for youth (CASES) and by providing additional psychometric data to help better operationalize empathy measurements and standardize CASES for further use.

## Method

### Participants

The Jintan Child Cohort Study is an ongoing longitudinal study made up of three waves. This Cohort Study looks at early health factors in relationship to later physical and psychosocial wellbeing of children and adolescents, including cognitive, emotional, and behavioral outcomes in a representative sample of children living in Jintan city [15]. This study explores the data retrieved in wave 2. In the present study, the children in 6th grade at the time of data collection completed the cognitive, affective, somatic empathy scale, as well as other related questionnaires. The total sample included 448 males (52.09%) and 412 females (47.91%), for a total of N = 860 participants (mean age = 11.54±.64). The majority of the participants lived in urban (40.58%) and suburban areas (41.74%), while 17.67% lived in rural areas. Approximately 58% of the families are middle class. More than 60% of the parents attended an occupational school or college. 90% of the fathers and 73% of the mothers were employed, and 20% of the parents held a professional job [16].

Signed consent forms for children’s participation were obtained from the parents. IRB approval was obtained from the University of Pennsylvania and the Ethical Committee for Research at Jintan Hospital in China.

### Instrument translation

Raine and Chen [11] developed the original CASES using 118 empathy questions, and selected 30 items to be included in the final version. It was designed to produce a three-factor model that encapsulated cognitive, affective and somatic forms of empathy and a two-factor model that had positive and negative valence. We received permission from Dr. Raine to use the original CASES for translation in this study. The first author, who is fluent in English and Chinese, led a team of three in the translating process, including a psychologist and a Masters- student in Education. Following the standard translation procedure suggested by Brislin [17], we first performed a forward translation of the original instrument into Mandarin Chinese. Then, a monolingual reviewer examined this Chinese version for incomprehensible or ambiguous words. We then back-translated this first Chinese version into English. This back-translated English version was compared to the original version to assess discrepancies and to determine whether the inconsistencies could be attributed to the Chinese forward-translation or the English back-translation. Errors in the forward- or back-translation processes required repeating the process again and, if necessary, taking the measure through a second back-translation.

### Measures

#### Callous-Unemotional traits (CU)

The Inventory of Callous–Unemotional Traits (ICU) is a 24 item self-report scale designed to assess CU traits such as limited empathy and a lack of guilt [18]. Examples from the scale are “I seem very cold and uncaring to others”, “I do not feel remorseful when I do something wrong”. Derived from the CU scale of the Antisocial Process Screening Device (APSD)[19], the ICU was developed to overcome reliability limitations of the APSD. Children responded to items on a four-point scale ranging from 0 (not at all true) to 3 (definitely true). The reliability and validity of the self-report version of the ICU has been supported in adolescent samples [20]. The current study utilized the total ICU score by summing all 24 items and the Cronbach alpha was .83.

#### IQ

IQ was assessed using the Chinese version and norms of The Wechsler Intelligence Scale for Children-IV (WISC-IV) [21] [22]. The most recent edition of the Wechsler tests for school-age children includes two composite scores: verbal IQ (VIQ) and performance IQ (PIQ) subtests. Verbal subtests are totaled to produce a VIQ score to reflect verbal skills and crystallized intelligence. Performance subtests are totaled to produce a PIQ score to indicate visual-spatial skills and fluid intelligence. All subtests are combined to produce a Full Scale IQ (FIQ), which is recognized as a clinically meaningful estimate of a child’s general cognitive abilities. Details of the IQ test were described elsewhere [22] [23].

### Statistical analysis

Analyses were conducted using SPSS version 24.0 and R. Mean, SD, skewness and kurtosis were calculated. To test internal consistency, Cronbach’s alpha coefficient was calculated.

To test the factor structure of the CASES in the Jintan Study, CFA with maximum likelihood estimation was conducted on the 30-item CASES. Missing values were handled using techniques with the assumption of missing data completely at random [24]. We compared one-factor (overall empathy—Model 1), two-factor (positive/negative valence—Model 2) and three-factor (cognitive, affective, somatic—Model 3) models. Model fit was assessed with two goodness-of-fit indices: Root Mean Squared Error of Approximation (RMSEA) index and the Comparative Fit Index (CFI). For this study, the following criteria were used to evaluate model fit: χ^2^/df < 3.0, CFI >.95, RMSEA < .06 and SRMR < .08, which suggest a good fit [25][26]. Chi-square χ^2^/df < 5.0, CFI > .90, RMSEA < .08 and SRMR < .10 suggest an adequate fit [27]. The χ^2^ difference test with the Satorra-Bentler scaling correction was used to test which of the models best fit the data. If the χ^2^ difference is significant, it suggests that the less constrained model fits the data better than the more constrained model as the model fit is significantly improved after some parameters are allowed to be freely estimated instead of being constrained (Schermelleh-Engel, Moosbrugger, & Müller, 2003).

We also used multigroup CFA to test invariance of this instrument across gender. We assessed a series of nested models with increasingly strict constraints on parameters to establish configural, metric, and scalar invariance [28]. Configural invariance is established by assessing the overall model fitness. Invariance at the metric and scalar level was evaluated using the χ^2^ difference test. For example, if the χ^2^ difference test is not significant moving from the configural to the metric level, then metric invariance is established.

Gender differences were tested using Student’s t test as it has been found that boys are less empathic than girls on average when using self-report questionnaires (Baez, 2017). The partial correlations between the scales of CASES and ICU, IQ were conducted to test construct validity whilst controlling for age.

## Results

### Factor structure of CASES

CFA with maximum likelihood estimation was used to examine the three first-order models. The fit indices of the models are presented in Table 1. Model 1 was a general empathy factor. The standardized factor loading were all significant and ranged from .34 to .68, with a mean loading of .52, which demonstrated that the items generally converged meaningfully to the scale.

**Table 1:** Model fit indices from the confirmatory factor analysis.

Model	χ^2^	df	χ^2^ /df	CFI	RMSEA (95% CI)	SRMR	Model comparison	Satorra-Bentler Scaled Δχ2	df	p
**1.One-factor model**	1531.11	399	3.84	.86	.057(.054, .060)	.052				
**2.Two-factor model**	1486.89	398	3.74	.87	.056 (.053, .059)	.051	**1vs2**	**44.22**	**1**	**< .001**
**3.Three-factor model**	1351.53	396	3.41	.88	.053 (.050, .056)	.048	**1vs3**	**179.59**	**3**	**< .001**

Model 2 consisted of the two factors of positive and negative valence. The standardized factor loadings were all significant and ranged from .34 to .70, with a mean loading of .54, demonstrating that the items generally converged meaningfully to the scale.

Model 3 consisted of the three factors: cognitive, affective and somatic aspects of empathy. The standardized factor loadings were all significant and range from .34 to .68, with a mean loading of .54, demonstrating that the items generally converged meaningfully to the scale.

The overall results suggest that three models have χ2/df< 5.0, RMSEA < .06 SRMR < .08, and CFI was .86∼.88, demonstrating all three models was adequate, but not optimal because CFI<0.90. The RMSEA of the three-factor model improved to .053, and the CFI improved to .88, suggesting a satisfactory model fit [25–27].

In addition, A two-factor structure (positive-negative empathy, Model 2) was found to have a significantly better fit, compared to the one-factor model (Δχ^2^ (1) = 44.22, p < .001). The three-factor structure (cognitive-affective-somatic empathy, Model 3) was also found to have a significantly better fit, compared to the one-factor model (Δχ^2^ (3) = 179.59, p < .001).

### Invariance of factor structure across gender

Measurement invariance analyses were conducted to investigate factor structure similarity across gender. Results of the fit indices obtained from testing three levels of invariance for the factor models are shown in Table 2. For the 3-factor model (cognitive, affective, and somatic), configural invariance was met because the overall model fitness was acceptable. Metric invariance was established as the model fitness is marginally non-significant (p = .047) when the factor loadings were constrained to be the same across gender. However, when constraining item intercepts to be equal across gender, there was a significant difference in the intercepts between boys and girls (p < .001).

**Table 2:** Fit indices for gender invariance testing for the three factor model (cognitive, affective, somatic).

Invariance level	χ^2^	df	CFI	RMSEA (95% CI)	Model comparison	Satorra-Bentler Scaled Δχ^2^	df	p
**1. Configural**	1879.10	792	.87	.056 (.053, .060)				
**2. Metric**	1919.50	819	.86	.056 (.053, .059)	1 vs. 2	40.36	27	.047
**3. Scalar**	2056.60	846	.85	.058 (.055, .061)	2 vs. 3	137.09	27	< .001

### Descriptive statistics, internal reliability and scale inter-correlations

Table 3 summarizes descriptive statistics including means, SD, skewness, kurtosis, internal reliabilities, and range in item-total correlations for all scales. Reliability for the total empathy scale was high (.92) and satisfactory reliabilities were also obtained for the broader 10 or 15-item domains of both positive and negative valence for cognitive, affective, and somatic empathy (coefficient α = .79 to .86). As sufficient normality requires a univariate skewness of less than 2 and a kurtosis of less than 7 [29], our data were normally distributed with skewness and kurtosis values of all CASES scales ranging from -.50 to -.02 and -.58 to -.18 respectively. S1 Table shows the correlation about empathy scales. All the subscales are highly correlated with empathy total score. Inter-correlations (r) between the three main domains were as follows: cognitive-affective (.70), cognitive-somatic (.63) and affective-somatic (.71). Positive and negative empathy were correlated (.79). All correlations were significant (p < .0001).

**Table 3:** Mean, SD, skewness, kurtosis, Cronbach’s alpha and range of item-total correlations for all CASES scales.

Scales (# items)	Mean	SD	Skewness	Kurtosis	Cronbach's Alpha	Item-total range
**Total**	37.02	12.21	-.21	-.25	.92	.32-.63
**Positive (15)**	19.14	6.30	-.31	-.37	.86	.32-.63
**Negative (15)**	17.80	6.46	-.14	-.30	.85	.41-.57
**Cognitive (10)**	12.34	4.76	-.22	-.58	.86	.49-.62
**Affective (10)**	13.70	4.24	-.50	-.18	.79	.30-.55
**Somatic (10)**	10 .90	4.59	-.02	-.51	.80	.36-.55

Total, total empathy scale; Positive, positive empathy scale; Negative, negative empathy scale; Cognitive, cognitive empathy scale; Affective, affective empathy scale; Somatic, somatic empathy scale.

### Sex differences (construct validity)

Results of comparisons between males and female, including means, standard deviations, and effect sizes, are show in Table 4. As expected, females had higher empathy scores than males. Comparisons were in all cases statistically significant for all forms of empathy, with the highest effect size being for negative empathy (d = .36).

**Table 4:** Sex differences in CASES empathy scores with means, SDs, and effect sizes (Cohen’s d).

Scales (# items)	Males (*n* = 448)	Females (*n* = 412)	T	*p*	d
**Total**	35.38(12.58)	38.79(11.56)	4.13	.000	.28
**Positive (15)**	18.67(6.65)	19.76(5.93)	2.56	.011	.17
**Negative (15)**	16.71(6.55)	19.02(6.22)	5.30	.000	.36
**Cognitive (10)**	11.99(4.96)	12.81(4.56)	2.49	.013	.17
**Affective (10)**	13.03(4.34)	14.40(4.07)	4.77	.000	.33
**Somatic (10)**	10.36(4.67)	11.59(4.46)	3.94	.000	.27

Total, total empathy scale; Positive, positive empathy scale; Negative, negative empathy scale; Cognitive, cognitive empathy scale; Affective, affective empathy scale; Somatic, somatic empathy scale.

### Callous-unemotional traits (validity)

All the correlations between child reported callous-unemotional traits and empathy measures were significant (p < .05) and in the direction of lower empathy being associated with high CU traits. The “ICU total score” column in Table 5 shows the correlations ranged from -.23 (CU traits and Somatic Empathy) to -.39 (CU traits and Positive Empathy).

**Table 5:** Associations between CASES empathy scales, ICU and IQ.

	ICU total score	Verbal IQ	Performance IQ	Full IQ
**Total**	-0.35***	.28***	.11*	.25***
**Positive (15)**	-0.39***	.31***	.15***	.29***
**Negative (15)**	-0.31***	.21***	.10*	.19***
**Cognitive (10)**	-0.32***	.29***	.13**	.26***
**Affective (10)**	-0.38***	.27***	.11*	.23***
**Somatic (10)**	-0.23***	.17***	.04	.14**

Total, total empathy scale; Positive, positive empathy scale; Negative, negative empathy scale; Cognitive, cognitive empathy scale; Affective, affective empathy scale; Somatic, somatic empathy scale. ICU, Callous-Unemotional traits. All the correlation was controlling for age.

***p < .001

**p < .01

*p < .05

### IQ (construct validity)

As indicated in Table 5, relationships between CASES and IQ measures were positive and significant; Low empathy was associated with lower IQ.

## Discussion

The purpose of this study was to assess the validity and reliability of the Chinese version of the CASES for assessment of children’s cognitive, affective, and somatic empathy. Results of our CFA support a three-factor model consisting of cognitive, affective, and somatic empathy and a two-factor model consisting of positive and negative aspects of empathy, with both sets of results producing satisfactory model fits. Acceptable reliability indices, including Cronbach’s alpha and item-total correlations, were obtained for each of the three main factors as well as for positive and negative empathy. Construct validity was supported by results indicating that females scored significantly higher than males, with lower empathy being associated with lower IQ. Construct validity was also reflected in findings showing that children with lower empathy also showed more CU attributes. This study utilized a relatively large sample that is representative of both rural and urban populations, lending support to the generalizability of these findings.

The CFA results revealed that the Chinese CASES possesses a stable three-factor structure with satisfactory loadings on each factor. This is consistent with that of the original English version [11]. Furthermore, multigroup analysis was conducted by gender to assess measurement invariance across groups, with configural invariance for gender being established. Metric invariance was found to be of marginal significance. Configural invariance and metric invariance allow us to make the assumption that the same factor structure with equivalent factor loadings can be built for both groups [28]. However, scalar invariance failed to be established for gender. That is, the factor structure for each group had variant intercepts [28]. Thus, no group comparisons can be made among the common factor scores [28]. Future studies using larger and more representative samples are needed to investigate whether scalar invariance can be established and to explore invariance across other demographic groups, such as those relating to age, SES, and clinical conditions.

We found a negative association between different aspects of empathy and total ICU score, which is consistent with past studies and recent reviews [1,30,31]. Deficits in affect regulation have been implicated in child psychopathology [32,33]. Similarly, it has been posited that callousness may be a fundamental contributor to future violent and antisocial behavior [34,35]. Items in the Affective Empathy CASES scale, such as “I would feel angry if I saw a man hitting a defenseless woman” and “Seeing a man pointing a gun at an unarmed person would make me feel frightened,” tap into a respondent’s empathic reaction to violent and antisocial behaviors. In addition, children with CU traits tend to show a lower heart rate change when watching videos with emotional evocative scenarios [36,37]. In this context, items in the Somatic Empathy CASES scale, such as “My heart beats faster watching an action-adventure movie” and “My heart beats faster when I see a scary TV show”, reflect a cardiovascular component to empathy which can be of value in future studies. Parsing the relationship between CU traits and cognitive empathy is difficult in children younger than 8 years, as young children generally do not have the capacity to fully understand and verbalize their internal states [38]. However, the children in the current study were all older than 9 years, lending confidence to our findings of a negative association between cognitive empathy and CU traits, a finding supported by other [39,40].

Another aspect of construct validity was evidenced by lower empathy being associated with lower IQ. While several previous studies have found no direct linkage between empathy and IQ, they have found that emotional intelligence has been positively correlated to both IQ (Fiori & Antonakis, 2012) and empathy (Stanley & Bhuvaneswari, 2016). Although we did not measure emotional intelligence, it is possible that the positive association between empathy and IQ that was found in this study could be mediated, at least in part, by emotional intelligence. It is also noteworthy that our findings of gender differences across all aspects of empathy—also evidence of construct validity—is consistent with other well-established findings that girls are more empathic than boys, on average [41,42].

Finally, the successful translation of CASES into Chinese has potentially important implications for child developmental and mental health outcome research in Chinese populations. Over the past few decades, China has experienced an increase in numerous social and public health problems, including gambling [43], cigarette use [44], and illicit drug use [45]. Rapid economic growth, modernization, and social change have been accompanied by an increase in awareness of mental illness and treatment needs in China (State Council of the People’s Republic of China, 2011; Cyranoski, 2010) particularly for Chinese adolescents [46]. To better understand rates of psychological disturbance in Chinese youth, recent efforts have sought to identify cultural contextual factors that may increase vulnerability, such as stress related to academic performance and its effect on depression and suicidality [47]. Our translation of the CASES would appear to fulfill such a need, although further application in larger samples is warranted to better understand whether it has any predictive utility.

## Limitations

Although we believe that findings from our novel analysis are potentially important for advancing our understanding of empathy in child developmental and mental health outcomes, some limitations exist. For example, this study is restricted to children no older than 12 years. Future research is needed to extend our understanding of empathy measurement in different age groups. This is an appropriate next step given that CASES is designed for a wide range of ages and development stages, from children to adults.

## Conclusions

This study is the first psychometric analysis of the only non-English translation of CASES to date. Results of CFAs reflect a successful translation from its original English version into Chinese which is consistent with reliability and validity analyses from Raine & Chen [11], thereby establishing for the first time cross-cultural validity for this scale. Our findings support previous research advocating a three-factor structure for operationalizing empathy into cognitive, affective, and somatic components [4,6–8], which could help clarify discrepancies in the published literature on associations between empathy and children developmental and mental health outcomes. The use of culturally appropriate, validated measures to assess potential contributions to child developmental and mental health outcomes and future aberrant behaviors could be a boon for the creation of prevention and risk-reduction efforts as well as for advancing our understanding of neurobiological deficits that underlie psychiatric disturbance.

## Supporting information

S1 Table Correlation in empathy scales. (DOCX)
