# Supplementary material for: The Chinese version of the cognitive, affective, and somatic empathy scale for children: Validation, gender invariance and associated factors
Source: PLoS One. 2018 May 7;13(5):e0195268. doi: 10.1371/journal.pone.0195268 (PMC5937997; doi:10.1371/journal.pone.0195268)
Supplement: S1 Table — (DOCX) [file pone.0195268.s001.docx]

**Table S1. Correlation in empathy scales.**

|  | **Total** | **Positive (15)** | **Negative (15)** | **Cognitive (10)** | **Affective (10)** | **Somatic (10)** |  |
| --- | --- | --- | --- | --- | --- | --- | --- |
| **Total** | 1 |  |  |  |  |  |  |
| **Positive (15)** | .95^**^ | 1 |  |  |  |  |  |
| **Negative (15)** | .95^**^ | .79^**^ | 1 |  |  |  |  |
| **Cognitive (10)** | .88^**^ | .85^**^ | .83^**^ | 1 |  |  |  |
| **Affective (10)** | .90^**^ | .87^**^ | .85^**^ | .70^**^ | 1 |  |  |
| **Somatic (10)** | .89^**^ | .82^**^ | .87^**^ | .63^**^ | .71^**^ | 1 |  |

*Note.* Total, total empathy scale; Positive, positive empathy scale; Negative, negative empathy scale; Cognitive, cognitive empathy scale; Affective, affective empathy scale; Somatic, somatic empathy scale.
